# Antimicrobial peptides from *Rana* [*Lithobates*] *catesbeiana*: Gene structure and bioinformatic identification of novel forms from tadpoles

**DOI:** 10.1038/s41598-018-38442-1

**Published:** 2019-02-06

**Authors:** Caren C. Helbing, S. Austin Hammond, Shireen H. Jackman, Simon Houston, René L. Warren, Caroline E. Cameron, Inanç Birol

**Affiliations:** 1 Department of Biochemistry and Microbiology, University of Victoria, Victoria, British Columbia V8P 5C2 Canada; 2 Canada’s Michael Smith Genome Sciences Centre, BC Cancer Agency, Vancouver, BC V5Z 4S6 Canada

## Abstract

Antimicrobial peptides (AMPs) exhibit broad-spectrum antimicrobial activity, and have promise as new therapeutic agents. While the adult North American bullfrog (*Rana* [*Lithobates*] *catesbeiana*) is a prolific source of high-potency AMPs, the aquatic tadpole represents a relatively untapped source for new AMP discovery. The recent publication of the bullfrog genome and transcriptomic resources provides an opportune bridge between known AMPs and bioinformatics-based AMP discovery. The objective of the present study was to identify novel AMPs with therapeutic potential using a combined bioinformatics and wet lab-based approach. In the present study, we identified seven novel AMP precursor-encoding transcripts expressed in the tadpole. Comparison of their amino acid sequences with known AMPs revealed evidence of mature peptide sequence conservation with variation in the prepro sequence. Two mature peptide sequences were unique and demonstrated bacteriostatic and bactericidal activity against Mycobacteria but not Gram-negative or Gram-positive bacteria. Nine known and seven novel AMP-encoding transcripts were detected in premetamorphic tadpole back skin, olfactory epithelium, liver, and/or tail fin. Treatment of tadpoles with 10 nM 3,5,3′-triiodothyronine for 48 h did not affect transcript abundance in the back skin, and had limited impact on these transcripts in the other three tissues. Gene mapping revealed considerable diversity in size (1.6–15 kbp) and exon number (one to four) of AMP-encoding genes with clear evidence of alternative splicing leading to both prepro and mature amino acid sequence diversity. These findings verify the accuracy and utility of the bullfrog genome assembly, and set a firm foundation for bioinformatics-based AMP discovery.

## Introduction

Antibiotic resistance among bacterial pathogens that cause prevalent global diseases has emerged as one of the most critical public threats facing the world today^1–3^. An analysis conducted by the Centers for Disease Control and Prevention estimates that at least 23,000 deaths in the United States each year are attributed to infections caused by antibiotic-resistant organisms^1^. In 2015, the World Health Organization endorsed a global action plan to combat antimicrobial resistance with strategic objectives that include optimizing the use of antimicrobial agents and sustainable investment in countering antimicrobial resistance^4^. Consequently, discovery and development of alternative antimicrobials is an urgent global need. As an alternative to traditional antibiotic therapy, antimicrobial peptides (AMPs) are garnering interest as potential therapeutics^5^. AMPs are a diverse class of peptides produced by all multicellular organisms as a defense against a broad spectrum of pathogens including bacteria, fungi, and viruses, and are considered central components of the innate immune system^6–8^.

Although overall AMPs exhibit remarkable sequence and structural diversity, commonalities include a typical length less than 100 amino acids, a positive net charge, and membership in one of four distinct groups based on their secondary structures: β-strand, α-helix, extended coil, and loop. Of these groups, α-helix AMPs are the most common and best characterized^6,9,10^. The cationic nature and the distribution of hydrophobic residues in AMPs enable these peptides to interact with and neutralize pathogens^6,11^.

AMP structure may show variability across the tree of life^12^. Amphibian AMPs are generally composed of an N-terminal signal peptide presequence, an adjacent prosequence that functions to maintain the AMP in an inactive conformation, and a C-terminal mature peptide sequence. All eukaryotic AMPs are synthesized as precursors that are proteolytically processed by propeptide convertases to yield active, mature peptides^8,13–15^. While AMP signal peptides and prosequences are typically conserved within families, the mature peptide sequences vary considerably, and constitute the functional portion of the antimicrobial peptide^8^. These characteristics can be exploited to identify and characterize novel AMPs from a large dataset^10^. Furthermore, because of the multifaceted mechanisms of antimicrobial action employed by AMPs, such as destruction of microbial membranes^16^, inhibition of macromolecule synthesis^17^, and peptide-induced modulation of the immune system^18^, microbes are less likely to develop resistance against these peptides than against conventional antibiotics. Several AMPs are currently used in a clinical setting, and many more AMPs are undergoing clinical trials to ascertain their therapeutic potential^11^.

The predominant approaches for isolating new AMPs involves chromatography- and/or mass spectrometry-based analyses of protein samples from body fluids or tissues in combination with antimicrobial assays, peptide sequencing, and *de novo* peptide synthesis. However, context-specific protein expression, the cost of implementation, and low throughput experimentation associated with traditional AMP identification methods that employ analytical chemistry have hindered AMP discovery progress. This emphasizes the need to develop an alternative approach for the identification of novel AMPs with therapeutic potential.

Adult frog skin is an abundant source of AMPs due to specialized granular glands in the dermis that synthesize and store these peptides, which are secreted onto the skin surface at the first sign of injury or microbial challenge^6,9,19^. From an evolutionary survival perspective, this rich repertoire of AMPs within frog skin is a beneficial adaptation to their wet and muddy environments where pathogens are plentiful. As of this writing, the curated Antimicrobial Peptide Database (APD)^20^ contains sequences for 978 active peptides originating from frog skin (out of 1043 amphibian peptides). This represents 34% of the AMP database compendium, which includes peptide sequences derived from six kingdoms including bacteria, archaea, protists, fungi, plants, and animals as well as some synthetic peptides (http://aps.unmc.edu/AP/main.php). Furthermore, the utility and efficacy of some frog AMPs as potential therapeutics has been demonstrated previously^21–23^.

AMP secretion is not just limited to amphibians, nor limited to the skin or a specific developmental stage. For example, liver-expressed antimicrobial peptides (LEAPs) are highly abundant in the liver and midgut and, in humans and fish, are secreted into the blood^24–26^. As amphibians, most frogs experience life in two distinct postembryonic forms: as a free-living aquatic larval tadpole and as an air-breathing terrestrial frog. The demands on the innate immune system differ as the types of pathogens living in each environment can differ substantially. Therefore, there is an opportunity to identify novel AMPs expressed in the larval stage. Tadpole-specific studies conducted to date have focused on testing natural skin secretions collected from a mixture of different aged tadpoles after immersion in or injection of norephinephrine. This established that these skin secretions could defend against parasitic worm infection and survival^27^. Using mass spectrometry, Woodhams and coworkers^28^ compared the norephinephrine-induced skin secretions of 17 frog species and found that Ranatuerin-2, -4, -6, -7, -8, and -9, Palustrin-2CBa, Bradykinin, Temporin-1P, and Ranalexin were the most abundant peptides. Generally, tadpoles had a lower proportion of AMPs relative to adults, but their profiles are distinct from each other^28^. Of these, Ranatuerin-2, -7, -8, -9, and Ranalexin were found even in the absence of norephinephrine induction^28^. An interesting finding was that tadpoles with longer larval periods, such as that of *R*. *catesbeiana*, produced a greater AMP defense response than tadpoles with short larval periods showing differential investment in the innate immune response at this aquatic developmental phase^28^.

Herein, we demonstrate the initial development of a bioinformatics approach for the identification and characterization of putative AMPs based on peptide homology. For this, we used a manually curated AMP sequence database to search the rich genomic resources that we have built for the North American bullfrog, *Rana* (*Lithobates*) *catesbeiana*^29^. We identify two novel bullfrog AMPs that demonstrate antimicrobial activity *via* an established microtiter broth dilution method^30^. Through computational methods applied to transcriptomics and genome data, we examine the expression profile and gene structures of twenty AMP-encoding transcripts, sixteen of which are found in tadpole tissues. This work sets the foundation for further large-scale, high throughput analysis of the recently published bullfrog genome^29^ to enable the identification and characterization of additional peptides with antimicrobial activity.

## Results

### Identification of putative AMP-encoding transcripts

A systematic stepwise *in silico* query of our Bullfrog Annotated Reference Transcriptome (BART^29^) database is outlined in the Methods section and resulted in the identification of seven *R*. *catesbeiana* transcripts encoding “novel” precursor AMPs (Table 1) and eleven “known” precursor AMPs (Suppl. Table 1). The translated “novel” precursor AMPs include a trypsin cleavage site (a common convertase cleavage site in AMPs) and, apart from one sequence that begins with a valine (HP3), all have a methionine at the N-terminus (Table 1). Further, the putative mature peptides all possess a predicted net positive charge at neutral pH, are between 19 and 33 AA in length, and have isoelectric points (pI) between 8.0 and 10.9 (Table 1). All of these physicochemical properties are consistent with those of known AMPs^20^.

**Table 1:** Characteristics of putative AMP sequences identified through bioinformatic analysis of bullfrog tadpole RNA-seq data.

Preprosequence	Putative Mature Peptide				
	Sequence	Net Charge	MW	pI	Peptide ID
MFTMKKSLLLLFFLGTISLSLCEQERNADDDQGEVIEQKVKR	AFLSTVKNTLTNVAGTMIDTFKCKITGVC	+2	3077.7	8.6	**HP2**
VLLYLIITVSFPRRDANDEDGGEVTKEVVKR	SLSGCWTKSFPRKPCLRNR	+5	2236.6	10.9	**HP3**
MSSFCEITNVALTISLSSPRRGADEEEGNGEKEIKR	SMLSVLKNLGKVGLGFVACKINKQC	+4	2651.3	9.6	**HP4**
MTQSTQKWFKTKYWRVRNRPAMSPDLNPIEHLWRDLKKVVGKR	NPSNLRALEELVKEECSEIPVERCKKLIYGYRK	+1	3908.5	8.0	**HP5**
MRKRMTMRRMMKKKKSEKERRERGKR	MMRVMRRKTKVIWEKKDFIGLYSID	+4	3144.8	10.2	**HP6**
MFFMSSPRRDADEVKEVKR	GFLDIIKNLGKTFAGHMLDKIKCTIGTCPPSP	+2	3417.1	8.6	**HP8**
MITVSSPRRDADGDEGEVEEVKR	GFLDIIKDTGKEFAVKILNNLKCKLAGGCPP	+2	3304.0	8.6	**HP9**

Each peptide sequence is separated into the prepro sequence and the presumed mature peptide sequence. Computational predictions of net charge, molecular weight (MW), and isoelectric point (pI) of the mature peptide are shown.

### Examination of putative AMP protein sequences

**Figure 1:**
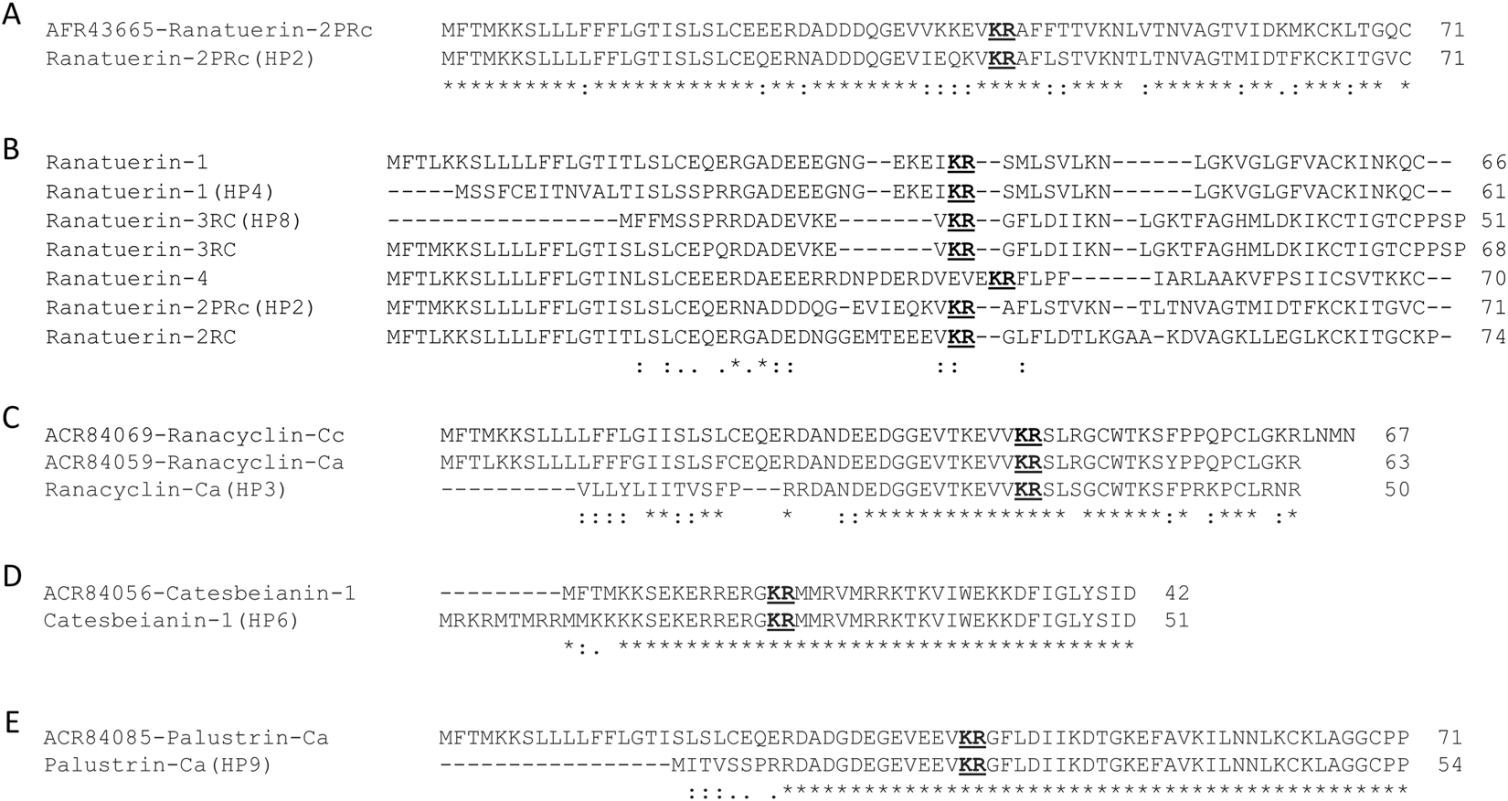
Clustal omega alignments of putative AMP precursor sequences with their closest known AMP matches. (**A**) Comparison of the *Pseudacris regilla* Ranatuerin-2PRc sequence (top) with the *R*. *catesbeiana* HP2 sequence (bottom). (**B**) Alignments of Ranatuerin precursor sequences from *R*. *catesbeiana*. (**C**) Alignments of Ranacyclin precursor sequences from *R*. *catesbeiana*. (**D**) Alignments of Catesbeianin-1 precursor sequences from *R*. *catesbeiana*. (**E**) Alignments of Palustrin-Ca precursor sequences from *R*. *catesbeiana*. The conserved proteolytic cleavage site is shown in bold and underlined. This cleavage site indicates the border for the N-terminal prepro sequence and the C-terminal mature sequence. The precursor peptide lengths are indicated to the right of each sequence. The dots represent conserved amino acid substitutions and asterisks indicate exact matches. Dashes were introduced to maximize sequence alignments. Further details regarding NCBI accession numbers are in Suppl. Table 1.

Blastx analyses of the seven transcripts identified protein sequence matches in the NCBI nr database ranging from 49–77% sequence identity (Table 2) and one sequence (HP5) had no notable match with any known AMP. Closer examination of the peptide sequences revealed that four of the predicted mature peptides (HP4, HP6, HP8, HP9) are identical to known AMPs (Table 2 and Fig. 1), while the corresponding prepro regions exhibit identities ranging from 65–76% (Table 2 and Fig. 1). The remaining two candidate AMP peptides (HP2 and HP3) exhibited 69% (HP2) and 68% (HP3) identity to their best-known AMP mature peptide sequence matches (Table 2). The HP2 and HP3 prepro sequences also show considerable divergence from their best-known AMP match (amino acid identities of 77% and 49%, respectively; Table 2). The HP2 sequence is the *R*. *catesbeiana* counterpart of the *Pseudacris regilla* Ranatuerin-2PRc sequence (Fig. 1A). When compared to the other known or putative AMP precursors, the Ranatuerin-2PRc (HP2) sequence exhibits a reasonable degree of sequence conservation with other Ranatuerins in the prepro sequence, but considerable divergence in the mature peptide (Fig. 1B). The putative mature peptide sequences of HP4 and HP8 are identical to the mature peptides of Ranatuerin-1 and Ranatuerin-3RC, respectively, but each has a distinct prepro sequence (Fig. 1B). The mature peptide region of HP3 is 68% identical to Ranacyclin-Ca, with substantial sequence divergence in the prepro sequence (41% identity; Fig. 1C). The mature peptide regions of HP6 and HP9 are identical to Catesbeianain-1 and Palustrin-Ca, respectively, but have divergent N terminal ends in the prepro sequences (Fig. 1D,E).

**Table 2:** Comparison of sequence identities (%) of the AMP candidates with their best-known AMP blastp matches over the entire sequence (precursor) or by prepro or mature sequences.

Peptide ID	Highest scoring blastp match	Sequence Identity (%)		
		Precursor	Prepro	Mature
HP2	Ranatuerin-2PRc	77	83	69
HP3	Ranacyclin-Ca	49	41	68
HP4	Ranatuerin-1	68	49	100
HP5	None	None	None	None
HP6	Catesbeianin-1	76	54	100
HP8	Ranatuerin-3RC	65	33	100
HP9	Palustrin-Ca	65	38	100

There was no AMP match with HP5.

**Figure 2:**
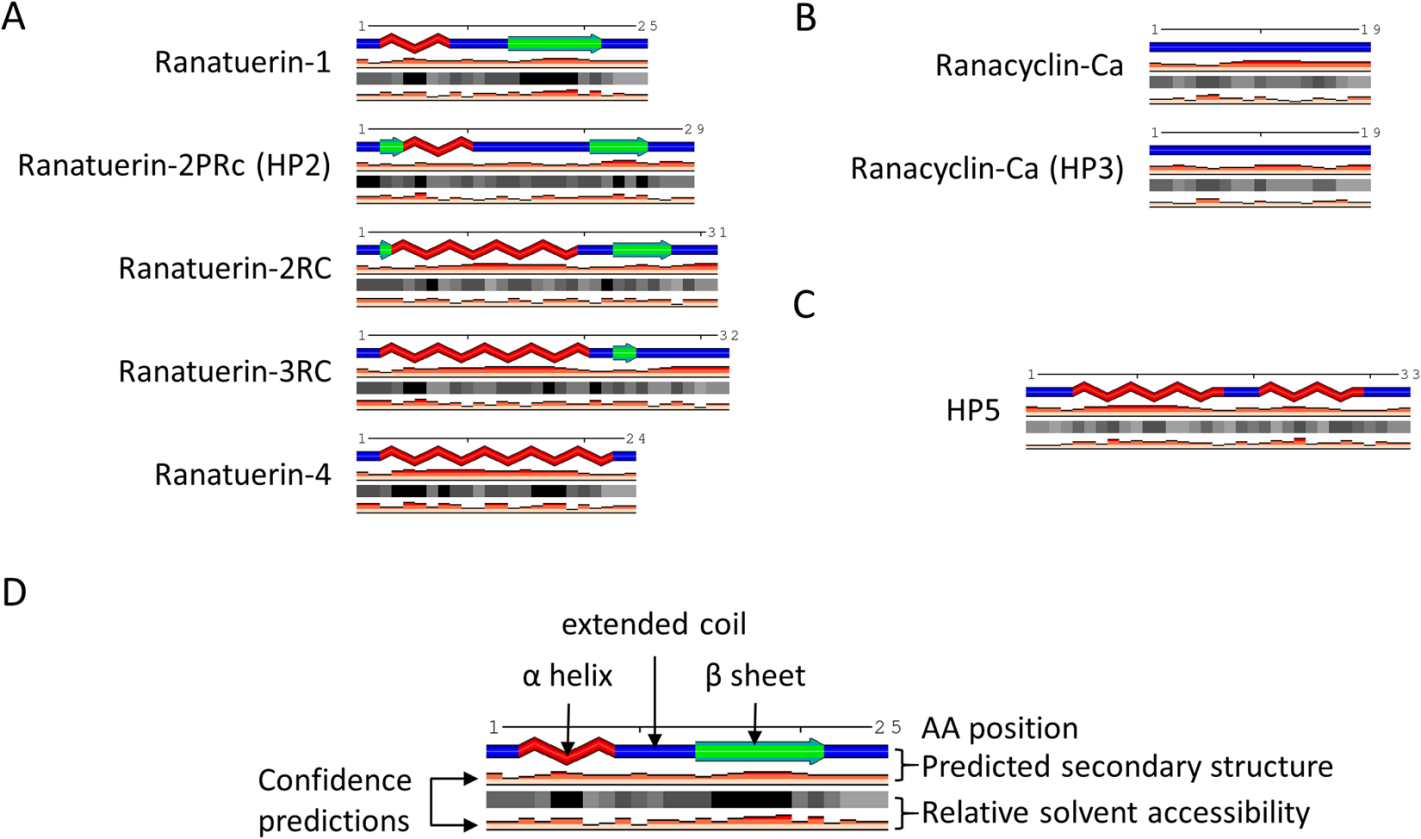
SABLE secondary structure prediction comparisons between the derived mature peptides of (**A**) HP2, (**B**) HP3, and (**C**) HP5 *versus* known mature AMP sequences. (**D**) Legend for the SABLE predictions with amino acid (AA) position indicated at the top, the predicted secondary structure in the middle and the relative solvent accessibility (RSA) at the bottom. Confidence predictions are below the predicted secondary structure and RSA. For the predicted secondary structure, red jagged lines, α helices; green arrows, β sheets; blue lines, extended coils. RSA is indicated by grey scale from black (0–9% RSA) to white (90–100% RSA) where each box represents an amino acid.

The secondary structure of the putative mature HP2 peptide contains an α-helix, extended coil, β-strand arrangement that resembles a mixture of Ranatuerin-1 and Ranatuerin-2RC secondary structure (Fig. 2A). The putative mature HP3 peptide is solely extended coil similar to Ranacyclin-Ca (Fig. 2B) while the putative mature HP5 peptide is comprised of two α-helices separated by a small extended coil (Fig. 2C).

### Microtiter broth dilution assays

HP2, HP3, and HP5 peptides are comprised of novel sequences that have not yet been described in the AMP literature. A common method for establishing antimicrobial activity of peptides is to perform microtiter broth assays. Microtiter broth dilution methods were implemented for determination of the minimum inhibitory concentration (MIC) and minimum bactericidal concentration (MBC). We tested HP3 and HP5 in addition to Ranatuerin-1/HP4 peptide as a positive control. We were unable to test HP2 because multiple peptide synthesis attempts failed. The human cathelicidin, LL-37^31^ was used as an additional positive control, and an unrelated similarly sized cationic peptide from the *T*. *pallidum* protein Tp0751 was used as a negative control. Given that a major mechanism used by AMPs is cell wall/membrane targeting^6^, we tested the potential AMPs against bacteria representing all three types of known cell envelope (Gram positive, negative, and the complex and unique mycobacterial cell wall/envelope).

Five bacterial species were tested, spanning Gram-negatives (*Escherichia coli* and *Pseudomonas aeruginosa*), Gram-positives (*Staphylococcus aureus* and *Streptococcus pyogenes*), and *Mycobacterium smegmatis* (neither a true Gram-positive nor Gram-negative). The Ranatuerin-1 peptide had some activity against *E*. *coli* and *S*. *aureus* (MIC: 48 and 97 μM, respectively) which is higher than previously reported^32^ and some bacteriocidal activity was observed (MBC: 12–48 and 97 μM, respectively). This peptide had no effect on *S*. *pyogenes* or *P*. *aeruginosa*. HP3 and HP5 displayed no inhibitory or bacteriostatic activity against *E*. *coli*, *S*. *aureus*, *S*. *pyogenes*, or *P*. *aeruginosa*. Except for the negative control peptide, all peptides had bacteriostatic activity against *M*. *smegmatis* (Table 3). Compared to Ranatuerin-1, HP3 had comparable bacteriostatic activity (MIC: 4–14 *versus* 2–12 µM; Table 3), and better bactericidal activity (MBC: 7–14 *versus* 3–48 µM; Table 3). HP5 exhibited weak bacteriostatic and bacteriocidal activity against *M*. *smegmatis* (Table 3).

**Table 3:** Minimum inhibitory concentrations (MIC) and minimum bactericidal concentrations (MBC) in μM against *M*. *smegmatis* for tested known and putative AMPs from a minimum of five (MIC) and three (MBC) independent experiments.

Peptide Name	μM	
	MIC	MBC
HP3	4–14	7–14
HP4/Ranatuerin-1	2–12	3–48
HP5	66	≥66
LL-37	0.4–2	1–28
Tp0751	—	—

LL-37 is a human cathelicidin positive control and Tp0751 is a negative control peptide from *T*. *pallidum*. “−” No effect observed.

### Expression of AMP-encoding transcripts in *R*. *catesbeiana* tadpole tissues

**Figure 3:**
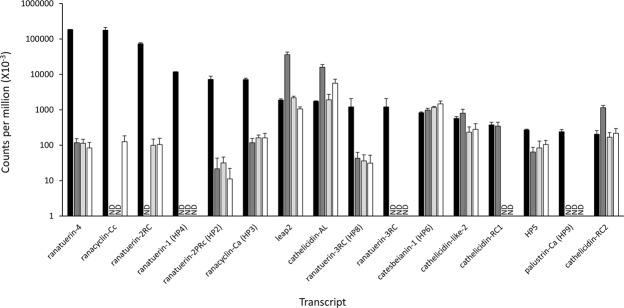
Putative and known AMP-encoding transcripts show differential expression in *R*. *catesbeiana* premetamorphic tadpole back skin, liver, olfactory epithelium, and tail fin. RNA-seq data representing transcripts encoding the indicated putative and known AMPs are shown for tadpole back skin (black bars, n = 3), liver (dark grey bars, n = 15), olfactory epithelium (light grey bars, n = 15), and tail fin (white bars, n = 15). Bars represent normalized median read counts per million and whiskers represent median absolute deviation. ND, not detected.

The abundance levels of twenty AMP-encoding transcripts (thirteen known and seven novel identified above; listed in Suppl. Table 1) were assessed in premetamorphic *R*. *catesbeiana* tadpole back skin, liver, olfactory epithelium, and tail fin using normalized RNA-seq data from previous studies^29,33,34^. Sixteen of these transcripts are in one or more of these tissues (Fig. 3). All of the indicated transcripts in Fig. 3 were sequence-verified from *R*. *catesbeiana* contigs including transcripts encoding Cathelicidin-AL (87% identity with *Amolops loloensis* precursor protein; Suppl. Fig. 1) and LEAP2 (88% identity with *Nanorana parkeri* predicted precursor protein; Suppl. Fig. 1). In several cases, the RNA-seq-derived sequences provided substantial improvements in length over transcript sequences already curated in GenBank (Suppl. Table 1).

All sixteen transcripts are present in the tadpole back skin, while 11 are in the liver and olfactory epithelium, and 12 in the tail fin (Fig. 3). The most abundant transcripts are *ranatuerin-4*, *ranacyclin-Cc*, *ranatuerin-2RC*, and *ranatuerin-1* (*HP4*) in the back skin; *leap2*, *cathelicidin-AL*, *cathelicidin-RC2*, and *catesbeianin-1* (*HP6*) in the liver; *leap2*, *cathelicidin-AL*, *catesbeianin-1* (*HP6*), and *cathelicidin-like-2* in the olfactory epithelium; and *cathelicidin-AL*, *catesbeianin-1* (*HP6*), *leap2*, and *cathelicidin-like-2* in the tail fin (Fig. 3). Of note are the transcripts that are not in these premetamorphic tadpole tissues such as *catesbeianin-1*, *ranacyclin-Ca*, *ranatuerin-1*, and *palustrin-Ca*. These transcripts, which are detected in adult frog skin^32^, are replaced by *catesbeianin-1* (*HP6*), *ranacyclin-Ca* (*HP3*), *ranatuerin-1* (*HP4*), *ranatuerin-3RC* (*HP8*), and *palustrin-Ca* (*HP9*) (Fig. 3).

**Figure 4:**
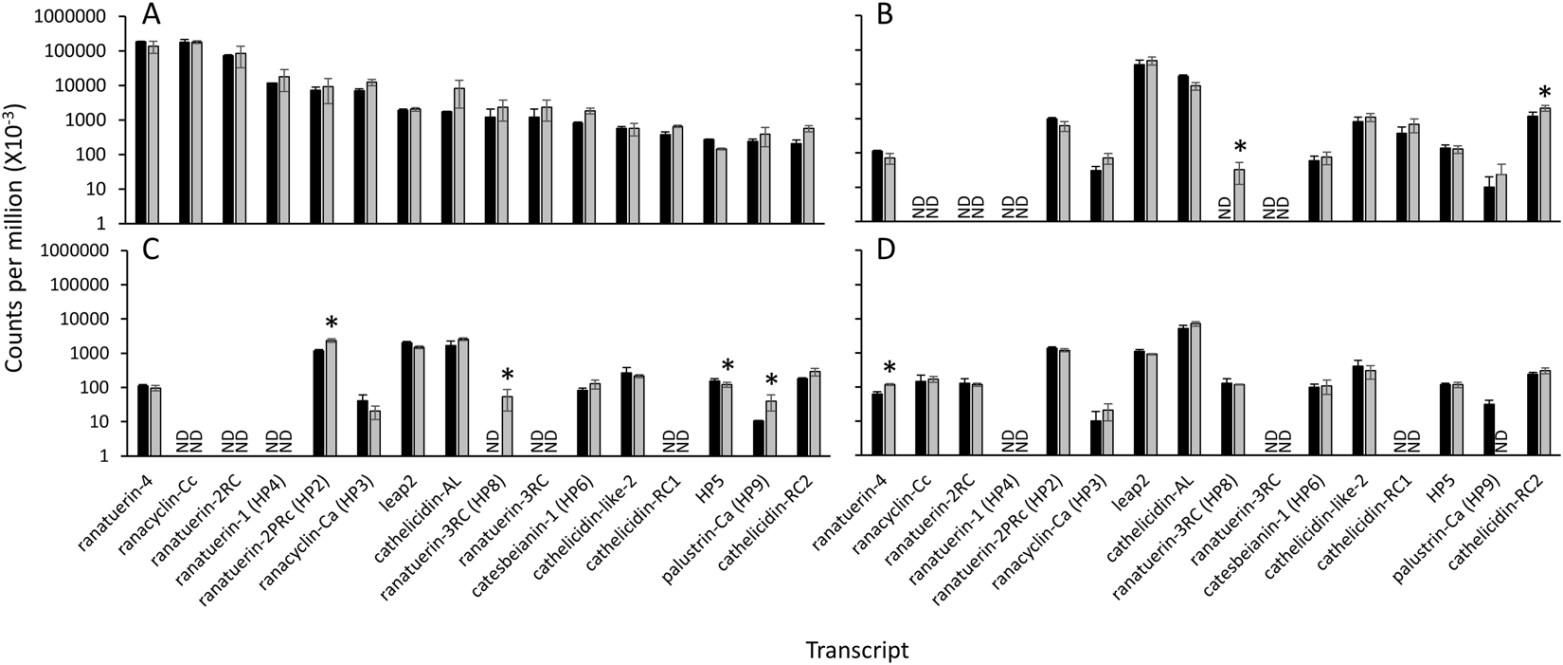
Putative and known AMP-encoding transcripts generally are not responsive to 10 nM T_3_ treatment of tadpoles. RNA-seq data representing transcripts encoding the indicated putative and known AMPs are shown for tadpole (**A**) back skin (n = 3), (**B**) liver (n = 5), (**C**) olfactory epithelium (n = 5), and (**D**) tail fin (n = 5) from vehicle controls (black bars) or tadpoles exposed to 10 nM T_3_ for 48 h (grey bars). Bars represent median read counts and whiskers represent median absolute deviation. The asterisk indicates statistical significance between treatments at p < 0.05. ND, not detected.

Previous work indicated that mRNAs encoding some AMPs increase from very low or undetectable levels in tadpoles to high levels in the frog as a consequence of thyroid hormone-dependent metamorphosis^35–38^. These determinations were done with either whole tadpole homogenates^35,37^ or skin^36,38^. We immersed premetamorphic tadpoles in 10 nM triiodothyronine (T_3_) for 48 h which precociously induces metamorphosis by altering tissue-specific gene expression programs^29,33,34^, and determined the abundance of the AMP-encoding transcripts (Fig. 4). None of these transcripts were responsive to T_3_ in the back skin (Fig. 4A). While the vast majority of transcripts also were not responsive to T_3_ treatment in the liver, olfactory epithelium, and tail fin (Fig. 4B–D), *ranatuerin-3RC* (*HP8*) transcripts appeared in the liver and olfactory epithelium (Fig. 4B,C). Significant increases in mRNA abundance were observed for *cathelicidin-RC2* (2-fold) in the liver; *ranatuerin-2PRc* (*HP2*) (2-fold) and *palustrin-Ca* (*HP9*) (4-fold) in the olfactory epithelium; and *ranatuerin-4* (2-fold) in the tail fin (Fig. 4B–D). A slight but significant decrease (1.3-fold) in *HP5* transcripts was observed in the olfactory epithelium (Fig. 4C). *Palustrin-Ca* (*HP9*) mRNA disappeared in the tail fin upon T_3_ treatment, but this was not significant (Fig. 4D).

### AMP-encoding gene structures in the bullfrog genome

**Figure 5:**
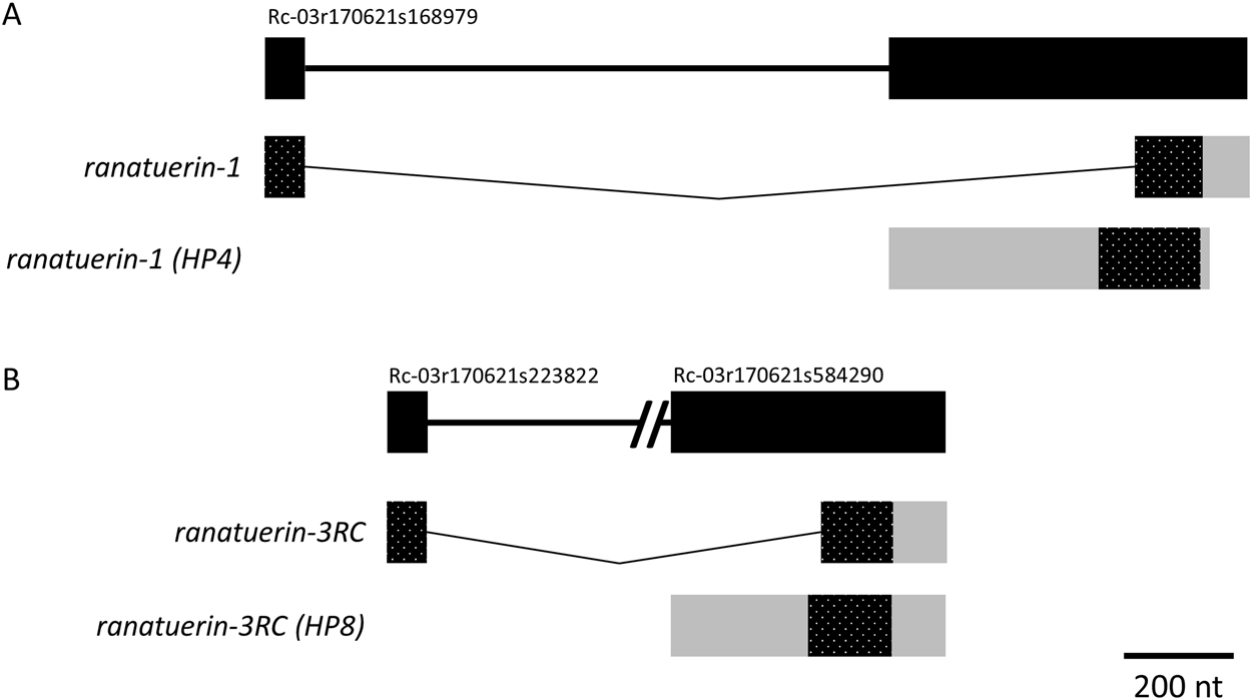
The *ranatuerin-1* and *ranatuerin-3RC* genes contain 2 exons and are alternatively spliced. The structure of the genes and derived transcripts encoding (**A**) Ranatuerin-1 and Ranatuerin-1 (HP4), and (**B**) Ranatuerin-3RC and Ranatuerin-3RC (HP8) are shown. The top illustration represents the corresponding gene drawn to the indicated scale with the exonic portions depicted as black rectangles and intronic regions depicted by the thick black line. The additional non-genic sequences flanking the indicated genes were present in all cases except where indicated. The NCBI v3.0 scaffold identifier from the bullfrog genome is indicated on the top left of each scaffold. Multiple scaffolds are indicated by a line break. Intronic regions are shown as thin lines that are spliced out in the labelled transcripts below the gene. The grey rectangles in the spliced transcript indicate the untranslated regions and the hatched rectangles indicate the open reading frame.

**Figure 6:**
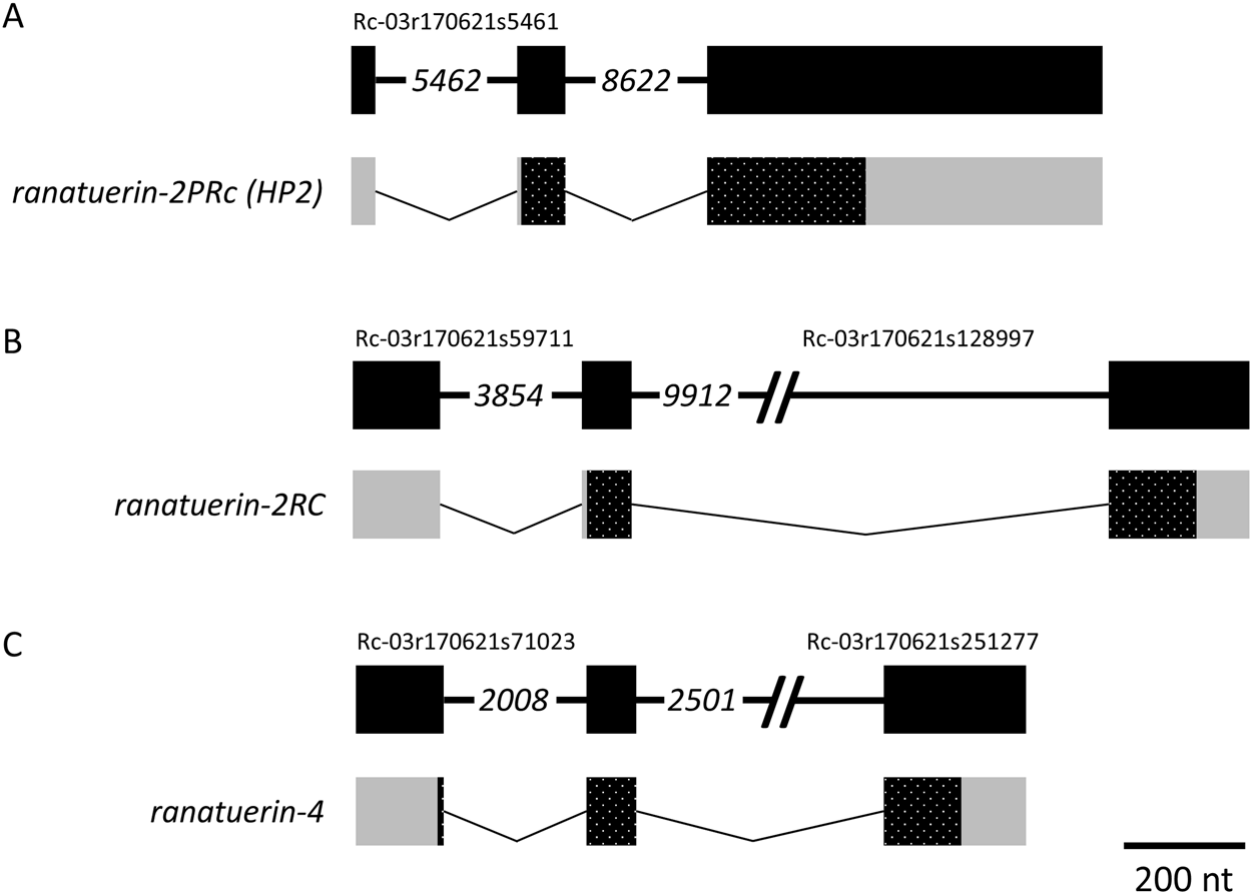
The *ranatuerin-2PRc* (*HP2*), *ranatuerin-2RC*, and *ranatuerin-4* genes are comprised of 3 exons. The structure of the genes and derived transcripts encoding (**A**) Ranatuerin-2PRc (HP2), (**B**) Ranatuerin-2RC, and (**C**) Ranatuerin-4 are shown. The illustrations are drawn to the indicated scale. The numbers in italics indicate the number of intervening base pairs where the intronic region was large. Refer to the Fig. 5 legend for more information.

**Figure 7:**
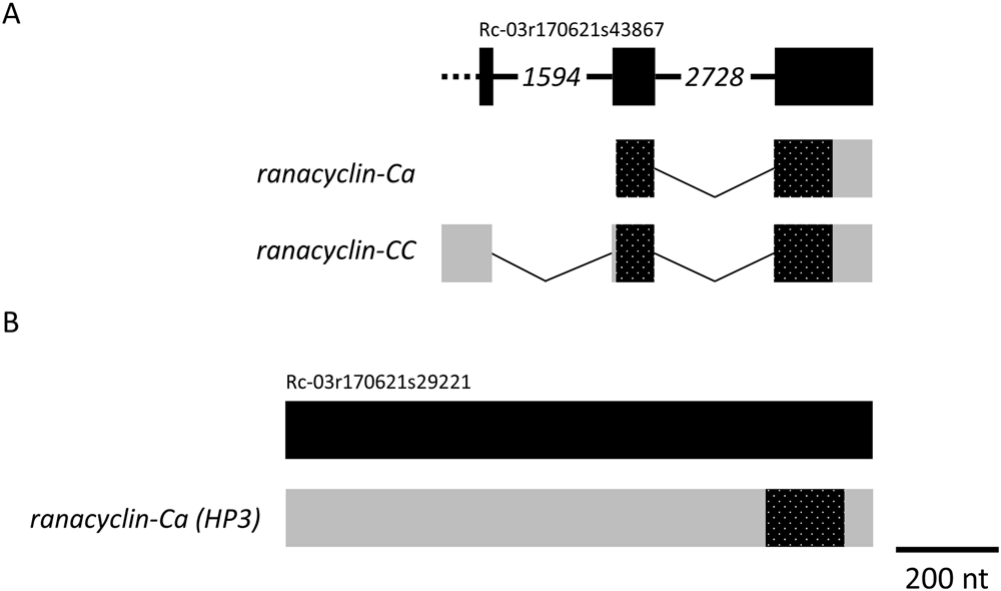
Two genes, one with 3 exons and the other with 1 exon, encode Ranacyclins. The structure of the genes and derived transcripts encoding (**A**) Ranacyclin-Ca and Ranacyclin-Cc, and (**B**) Ranacyclin-Ca (HP3) are shown. Refer to the Figs 5 and 6 legends for more information. The dotted line in “A” indicates that the 5′ end of the scaffold terminated prior to the available transcript information.

**Figure 8:**
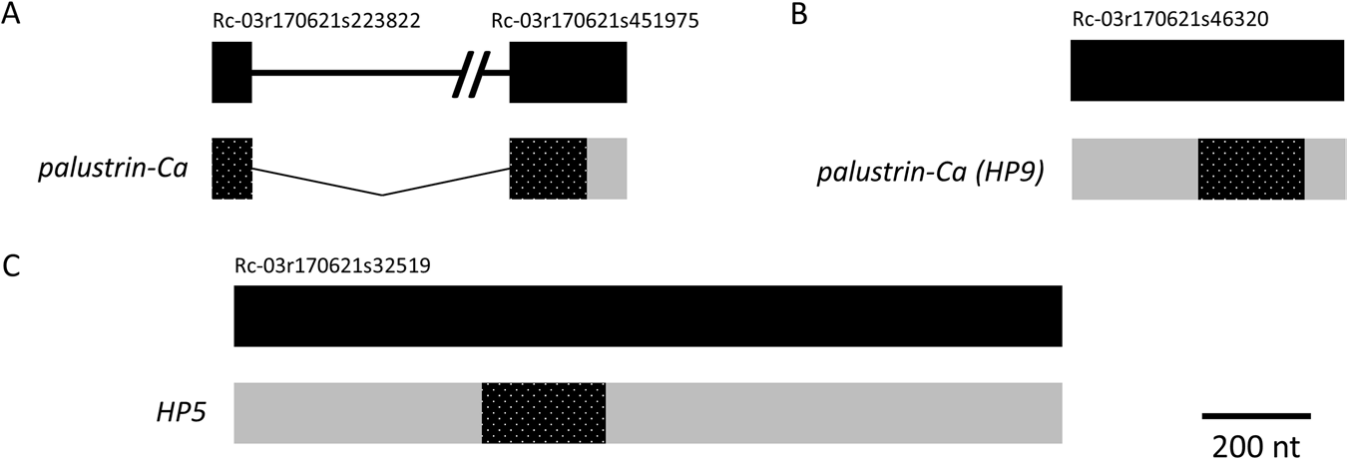
Palustrin-Ca is encoded by a 2 exon gene, while Palustrin-Ca (HP9) and HP5 are encoded by single exon genes. The structure of the genes and derived transcripts encoding (**A**) Palustrin-Ca, (**B**) Palustrin-Ca (HP9), and (**C**) HP5 are shown. Refer to the Figs 5 and 6 legends for more information.

**Figure 9:**
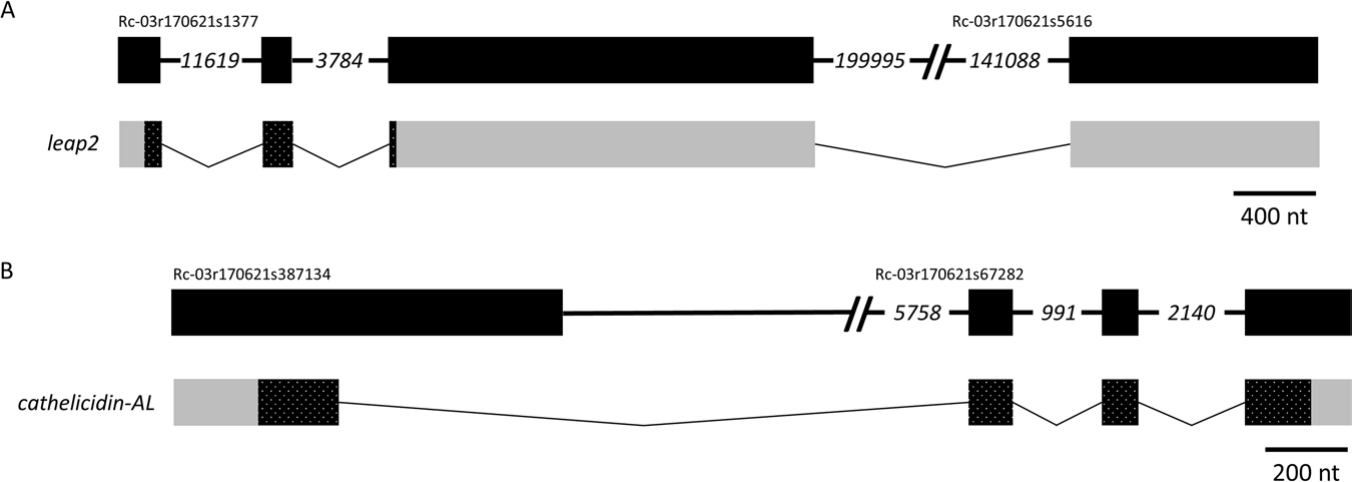
Both LEAP2 and Cathelicidin-AL are derived from four exons. The structure of the genes and derived transcripts encoding *R*. *catesbeiana* (**A**) LEAP2 and (**B**) Cathelicidin-AL are shown. Refer to the Figs 5 and 6 legends for more information.

It is possible that the new versions of the above transcripts were products of alternative gene splicing. Using our recently published draft bullfrog genome^29^, we used gmap and blastn to create gene models from the transcript sequences. A 1.6 kbp two-exon gene gives rise to *ranatuerin-1* and *ranatuerin-1* (*HP4*) through alternative splicing (Fig. 5A) and a similar two-exon gene structure gives rise to *ranatuerin-3RC* and *ranatuerin-3RC* (*HP8*) through alternative splicing (Fig. 5B). In contrast, the three ranatuerins, *ranatuerin-2PRc* (*HP2*), *ranatuerin-2RC*, and *ranatuerin-4*, are each derived from distinct three-exon genes that are much larger (e.g. 15 kbp for *ranatuerin-2PRc* (*HP2*); Fig. 6). The transcripts encoding Ranacyclin-Ca and Ranacyclin-CC come from the same 3-exon gene whereas the gene encoding ranacyclin-Ca (HP3) is comprised of a single exon on a different scaffold (Fig. 7). A similar relationship occurs for Palustrin-Ca. Here, the *palustrin-Ca* transcript is derived from two exons (Fig. 8A) and *palustrin-Ca* (*HP9*) from a different single exon (Fig. 8B) from the same gene. The gene encoding HP5 is comprised of a single exon (Fig. 8C). Finally, the *leap2* and *cathelicidin-AL* transcripts are examples derived from the splicing of four exons (Fig. 9). The fact that all assembled transcript sequences above align with the independently-derived bullfrog genome with canonical splice sites further supports the legitimacy of the identified AMP transcript sequences.

## Discussion

By utilizing known sequence homology and structural characteristics of AMPs from empirically validated peptides, we applied a bioinformatics approach to an assembled bullfrog tadpole reference transcriptome, and identified transcripts encoding putative novel AMPs and augmented the sequence information available for known AMPs. Historically, frogs are a rich source of AMPs. However, studies on larval tadpole stages have been limited, particularly those pertaining to gene expression studies.

The present study focused on the premetamorphic tadpole as an organism that is primarily dependent upon the innate immune system for microbial protection. Similar to what was observed in the frog, tadpole tissues express several AMPs with the greatest concentration in the back skin. Previous work in *Xenopus laevis* indicated that transcripts encoding magainin and “peptide with amino terminal glycine and carboxy terminal leucinamide” (PGLa) are not detected until metamorphic climax and into the frog stage^35^. The abundance of these mRNAs increase in whole premetamorphic tadpoles by prolonged immersion in 5 nM T_3_ for 7 d, inducing precocious metamorphosis^35^. Other studies established that mRNAs encoding Ranalexin in *R*. *catesbeiana*^36^, Brevinin-1SY in *R*. *sylvatica*^37^ and Preprotemporin in *R*. *ornativentris*^38^ generally transition from undetectable or very low levels in the tadpole through thyroid hormone-dependent metamorphosis to high levels in the frog. An induction of Preprotemporin-encoding mRNA upon injection of adult *R*. *ornativentris* with 2 × 10^−9^ M T_3_ was observed^38^. We were able to examine the abundance levels of twenty known and putative AMP-encoding transcripts of which sixteen were expressed in at least one of the four premetamorphic tadpole tissues in the present study. The vast majority of AMP-encoding transcripts were not affected by T_3_ treatment after 48 h and none were hormone-responsive in the back skin. It is difficult to compare the previous studies with our results due to the use of whole tadpole homogenates rather than specific tissues and/or the use of adults instead of tadpoles for T_3_ injection studies. It is possible that longer T_3_ exposure times may result in modulation of more AMP-encoding transcripts, but this remains to be determined. The data suggest that the metamorphosis-dependent change in AMP expression may be a later indirect thyroid hormone-dependent response leading to a resetting of the innate immune system coinciding with life transition.

The antimicrobial properties of Catesbeianin-1, Ranacyclin-Ca, Ranatuerin-1, Ranatuerin-3RC, and Palustrin-Ca have been known for some time^6–8^. The work reported herein presents the discovery that there can be diversity in their prepro sequences while retaining the mature peptide sequence of the respective AMP as a consequence of alternative splicing. An intriguing possibility is that the gene splice variants may be developmentally regulated as part of resetting the immune system during postembryonic development. The consequence of this shift embodies a change in prepro sequence rather than the mature peptide of the respective AMP. This may have regulatory consequences for peptide localization, processing and/or activation that have yet to be determined, and may possibly reflect a developmental shift in expressed activating proteases as well^39^. Further examination into the expression profiles of the splice variants during development and in a broader range of tissues is warranted.

While considerable efforts have been placed on phylogenetic comparisons of AMPs at the protein level (for e.g.^40,41^), much less is understood regarding the structure of the genes giving rise to AMP-encoding transcripts. The current study presents the first gene structure information of AMPs with known antimicrobial functionality in frogs, and we found a range of AMP gene structures represented. The four-exon gene structure observed in *R*. *catesbeiana cathelicidin* is conserved with the human LL37 *cathelicidin* gene on chromosome 3 (NCBI Accession NM_004345.4) while the *R*. *catesbeiana leap2* gene has four exons compared to three in fish and humans^26^. This apparent discrepancy may be due to the fourth exon comprised entirely of untranslated region (Fig. 9). As the *R*. *catesbeiana leap2* gene structure is currently composed of two scaffolds, we cannot definitively discount the possibility that the *leap2* transcript may be an assembly artifact. Further improvements to the bullfrog genome assembly will resolve this issue.

The Ranatuerin-encoding genes are subject to alternative splicing and possess two or three exons in *R*. *catesbeiana*, and the close relationship between Ranatuerins and Ranacyclins are reflected in the retention of the three-exon gene structure. Further, the diversity of AMP mature peptide sequences have been suggested to be a consequence of gene duplications from an ancestral gene^42^. The present study provides support for this in addition to alternative splicing as another mechanism for AMP diversity.

Two new mature AMP sequence candidates, in addition to Ranatuerin-1, demonstrated antimicrobial activity against *M*. *smegmatis*. Of particular note, HP3 and the established AMP, Ranatuerin-1, exhibited similar antimicrobial activity against this mycobacterial species. We used this species to establish proof-of-concept that our novel AMPs are active against Mycobacteria. While permeabilization of their highly complex and unique cell envelope is the suggested mode of AMP action, it is not clear whether this is sufficient for cytotoxicity or whether additional AMP interactions with intracellular components, such as DNA, is required^43^. Regardless, since all mycobacterial species have a similar cellular structure, demonstrating activity against a classic non-pathogenic species has provided us the evidence that it is worthwhile to next assess the activity of the novel AMPs against pathogenic species in future experiments.

While there is some variability within the activity results presented herein, the presented studies represent a first step in the process of designing optimized AMPs that exhibit improved consistency, reproducibility, stability, and enhanced activity^44^. Sequence analysis of the new AMP candidates revealed diversity within the prepro and the mature peptide sequences adding to the growing assortment of AMPs. The linkage of known AMP sequences to new prepro sequences opens up new possibilities for further AMP candidate discovery. Successful functional testing of AMPs identified *via* the bioinformatics methods used in the present study affirms the value of using a bioinformatics approach to mine the bullfrog genome, as described herein.

Because AMPs play a critical role in innate immunity^6,45^, further examination of the circumstances of their expression and factors that may disrupt their normal function could inform conservation efforts. Amphibians are experiencing drastically decreased population numbers worldwide due to infectious pathogens^45,46^. The interplay between AMPs and pathogens is an important determinant of host survival upon infection, and some amphibian AMPs are known, for example, to kill the chytrid fungus, *Batrachochytrium dendrobatidis*^47,48^. Resistance can be conferred by fungal secretion of a protease that cleaves and disrupts amphibian AMP function^47,48^ revealing the need for further investigations into the mechanisms of AMP regulation and their relationship to disease protection and pathogen evasion. In addition, continued investigations into the wealth of natural antibiotic compounds produced by amphibians will also undoubtedly result in further discovery of novel AMPs that may lead to the development of effective therapeutics for combatting the major and increasing global health threat of antibiotic resistance.

## Methods

### *In silico* prediction and characterization of putative antimicrobial peptides

Seven novel AMP candidates were initially identified from the bullfrog annotated reference transcriptome (BART version 3, NCBI TSA accession GFBS01000000)^29,34^ using the following three steps:

First, the BART transcript sequences, all of which were *de novo* assembled with Trans-ABySS^49^ from strand-specific RNA-Seq libraries^29^, were *in silico* translated using Transdecoder (-m 20 -S; version 2.0.1) (https://github.com/TransDecoder/TransDecoder) and complete predicted open reading frames up to 100 amino acids long were retained.

Second, Hidden Markov models (HMMs) representing the salient features of AMPs from 35 protein families were downloaded from the Collection of Antimicrobial Peptides database (http://www.camp.bicnirrh.res.in/pattern_hmm.php?q=HMM_fam; accessed March 3, 2016), and hmmer^50^ was used to identify BART peptide sequences with similarity to one or more HMM (default settings, significance considered at E < 0.001). These hits were then further refined using InterProScan^51^ default settings with the Pfam database^52^ of protein domain HMMs (version 29.0).

Third, candidate AMPs had to satisfy the following criteria: (1) the putative open reading frame began with a methionine or valine residue according to Virtual Ribosome 2.0^53^ analysis, and (2) the protein sequence contained a canonical propeptide convertase Lys-Arg (KR) cleavage site as determined by ExPASy Peptide Cutter (http://web.expasy.org/peptide_cutter). With the exception of one AMP candidate, all peptide sequences also had strong alignment to a known precursor AMP defined as an E-value score of <10^−4^ using blastx or blastp against the NCBI nr database. If the candidate AMP sequence had a full precursor alignment to a sequence in the NCBI nr database with identity and positivity scores of greater than 90%, then the sequence was considered “known”. A final set of seven “novel” and eleven “known” AMP-encoding *R*. *catesbeiana* transcripts were found from tadpole tissues (Suppl. Table 1). Two additional AMP sequences from previous studies on adult frogs were also examined in the present study (Suppl. Table 1). Final protein alignments were generated using Clustal Omega version 1.4.2 (http://www.ebi.ac.uk/Tools/msa/clustalo)^54^.

Secondary structures of the mature AMP peptides were assessed using SABLE Protein prediction (http://sable.cchmc.org/). The net charge, molecular weight, and isoelectric points (pI) of the mature peptides were determined using ExPASy ProtParam (https://web.expasy.org/protparam/).

### Gene expression analysis

The levels of twenty AMP-encoding *R*. *catesbeiana* transcripts (Suppl. Table 1) were determined in premetamorphic *R*. *catesbeiana* tadpole back skin, tail fin, olfactory epithelium, and liver tissues through RNA-seq data derived from previous studies of tadpole tissues^29,33,34^. Strand-specific mRNA libraries were constructed and sequenced *via* Illumina HiSeq and aligned to the BART reference transcriptome^29^ to generate counts. All RNA-seq experiments had comparable sequencing depth and were normalized to the total number of reads per sample. To normalize the counts, the number of reads were divided by the total number of reads in the corresponding sample and multiplied by 100 million.

### Gene structure determination

The longest cDNA sequence from each of twenty *R*. *catesbeiana* transcripts encoding AMPs (Suppl. Table 1) was used to query the high quality draft bullfrog genome (NCBI Accession number LIAG00000000, BioProject PRJNA285814)^29^ using gmap version 2017-04-13^55^. The relevant scaffolds are indicated in Suppl. Table 1.

### Microtiter broth dilution assays

To test for antimicrobial activity, HP3, HP4/Ranatuerin-1, and HP5, peptides were synthesized by GenScript (Piscataway, New Jersey, USA). HP2 was not tested because the service provider was unable to synthesize this peptide despite multiple attempts. An unrelated, similarly-sized peptide from the *Treponema pallidum* protein Tp0751^56^ was used as a negative control and the human cathelicidin, LL-37^31^, was included as a positive control. All peptides contained free termini and were tested in the oxidized state. Peptides were dissolved in filter-sterilized ultrapure water and tested for sterility by plating on non-selective agar plates followed by a 48 h incubation at 37 °C. Two-fold serial dilutions of each peptide were prepared to obtain a series corresponding to ten times the required testing concentrations (2560, 1280, 640, 320, 160, 80, 40, 20, 10, and 5 µg/mL)^30^.

Microtiter broth dilution methods were implemented for determination of the minimum inhibitory concentration (MIC) and minimum bactericidal concentration (MBC) of the four putative AMPs and the negative control peptide using procedures adapted from the R.E.W. Hancock Laboratory for cationic AMPs^30^ and the CLSI methods for dilution antimicrobial susceptibility tests^57^.

To assess antimicrobial activity across a diverse range of bacterial species, colonies were cultured overnight on Mueller Hinton agar plates (MHA; +5% sheep blood for *S*. *pyogenes*)^57^ from frozen glycerol stock. Bacteria tested include Gram-negative rods (*Escherichia coli*: ATCC 9723H; *Pseudomonas aeruginosa*: ATCC 10148), Gram-positive cocci (*Staphylococcus aureus*: ATCC 6538P; *Streptococcus pyogenes*: unknown strain, hospital isolate), and *Mycobacterium smegmatis* (MC^2^155; classified as neither Gram-positive nor Gram-negative). Bacterial suspensions were prepared by placing 3–5 morphologically similar colonies from the grown plate into sterile glass culture tubes containing 2 mL of Mueller Hinton Broth (MHB; +5% lysed horse blood for *S*. *pyogenes*)^57^. Microbial inoculums from bacterial suspensions were prepared through a spectrophotometric adjustment of turbidity to 0.08–0.1 at 600 nm to achieve a turbidity equivalent to that of a 0.5 McFarland standard (1–2 × 10^8^ CFU/mL)^30^. The standardized bacterial inoculums were then diluted in MHB to obtain final cell densities of approximately 5.0 × 10^5^ CFU/mL.

Ninety-six-well microtiter plates (Fisher Cat. No. CS003790; Nepean, Ontario, Canada) were prepared with 100 µL of *E*. *coli*, *P*. *aeruginosa*, *S*. *aureus*, *S*. *pyogenes*, or *M*. *smegmatis* bacterial suspension (5 × 10^5^ CFU/mL) dispensed into each well of columns 1 through 11. Eleven microliters of the 10x AMP dilution series for all four peptides were added to each well from column 1 (2560 µg/mL) to column 10 (5 µg/mL) in all plates. Column 11 functioned as a positive control for bacterial growth in the absence of AMPs. Column 12 in each plate contained 100 µL of MHB as a sterility control (+5% lysed horse blood for *S*. *pyogenes*)^57^. Plates were incubated at 37 °C for 16–24 within 15 min of adding the inoculum.

MIC values were visually determined by comparing the amount of bacterial growth (turbidity) in wells containing AMPs with growth in the control wells that did not contain any amount of peptide. MBC values were determined by plating the entire contents of the wells containing the peptide/bacteria mixture representing the MIC and the entire contents of the two preceding wells containing 2-fold and 4-fold more concentrated AMP dilutions/bacteria mixtures onto non-selective MHA plates, followed by incubation for 24 h at 37 °C.

### Ethical approval and informed consent

The study was conducted following animal protocol #2015–028 approved by the University of Victoria Animal Care Committee in accordance with the Canadian Council for Animal Care.

## Supplementary information

Supplementary Information

## Data Availability

DNA sequence information and RNA-seq data are available through NCBI as indicated in the text, figures, and tables.
