# Supplementary material for: Antimicrobial peptides from Rana [Lithobates] catesbeiana: Gene structure and bioinformatic identification of novel forms from tadpoles
Source: Sci Rep. 2019 Feb 6;9:1529. doi: 10.1038/s41598-018-38442-1 (PMC6365531; doi:10.1038/s41598-018-38442-1)
Supplement: Supplementary file 1 — Supplementary Information [file 41598_2018_38442_MOESM1_ESM.pdf]

Supplementary Material

Antimicrobial peptides from *Rana [Lithobates] catesbeiana*: Gene structure and bioinformatic identification of novel forms from tadpoles

Caren C. Helbing[1,*], S. Austin Hammond[2], Shireen H. Jackman[1], Simon Houston[1], René L. Warren[2], Caroline E. Cameron[1], and Inanç Birol[2]

[1] Department of Biochemistry and Microbiology, University of Victoria, Victoria, British Columbia, V8P 5C2, Canada

[2]Canada's Michael Smith Genome Sciences Centre, BC Cancer Agency, Vancouver, BC, Canada, V5Z 4S6, Canada

[*]Corresponding Author: Caren C. Helbing
E-mail: chelbing@uvic.ca
Phone: 1 (250) 721-6146
Fax: 1 (250) 721-8855

Suppl. Table 1. Transcript, protein, and gene characteristics of 20 known and putative AMPs evaluated within the present study.

	Closest NCBI nucleotide sequence		Closest NCBI precursor protein sequence			New *Rana catesbeiana* transcripts from BART			*Rana catesbeiana* gene information				
AMP name	Accession #	Length (nt)	Accession #	Length (AA)	Species	Accession #	Length (nt)	Length (AA)	Genome scaffold	Strand	Scaffold length (bp)	Range of overlap with query sequence by scaffold nt position	Exon #
catesbeianin-1	FJ830640	324	ACR84056	42	*Rana catesbeiana*	N/A[a]	N/A	N/A	None	None	None	None	None
catesbeianin-1 (HP6)	FJ830640	324	ACR84056	42	*Rana catesbeiana*	GFBS01479282	626	51	None	None	None	None	None
cathelicidin-like-2	KF766531	700	AHW58221	156	*Rana catesbeiana*	MH800186	753	155	None	None	None	None	None
cathelicidin-AL	JF923766	648	AEI69698	179	*Amolops loloensis*	MH800187	1019	181	Rc-03r170621s387134; Rc-03r170621s67282	+/-	1650/ 14834	1-400/ 9076-8969, 7977-7888, 5747-5490	4
cathelicidin-RC1	KF766530	677	AHW58220	151	*Rana catesbeiana*	MH800188	926	151	None	None	None	None	None
cathelicidin-RC2	KF766531	700	AHW58221	156	*Rana catesbeiana*	MH800189	753	155	None	None	None	None	None
HP5	None	None	None	None	*None*	GFBS01753449	1523	76	Rc-03r170621s32519	+	36533	3251-4772	1
leap2	XM_018563220	469	XP_018418722	81	*Nanorana parkeri*	MH800190	3507	81	Rc-03r170621s1377; Rc-03r170621s5616	+/+	279751/ 290780	62065-62169, 73789-73937, 77722-79756/ 141039-142257	4
palustrin-Ca	FJ830669	322	ACR84085	71	*Rana catesbeiana*	N/A	N/A	N/A	Rc-03r170621s223822; Rc-03r170621s451975	+/+	2611/ 1423	2144-2217/ 128-343	2
palustrin-Ca (HP9)	FJ830669	322	ACR84085	71	*Rana catesbeiana*	GFBS01150567	527	54	Rc-03r170621s46320[b]	+	24012	18718-19222	1
ranacyclin-Ca	FJ830643	311	ACR84059	63	*Rana catesbeiana*	N/A	N/A	N/A	Rc-03r170621s43867	+	25684	10079-10152, 12881-13068	2
ranacyclin-Ca (HP3)	FJ830643	311	ACR84059	63	*Rana catesbeiana*	GFBS01071740	1143	50[c]	Rc-03r170621s29221	-	41060	20482-19341	1
ranacyclin-Cc	FJ830653	296	ACR84069	67	*Rana catesbeiana*	GFBS01607132	524	62	Rc-03r170621s43867	-	25684	8419-8475, 10070-10152, 12881-	3

												13075	
ranatuerin-1	FJ842524	314	ACR46972	66	*Rana catesbeiana*	N/A	N/A	N/A	Rc-03r170621s168979	-	3446	2612-2539, 1247-1038	2
ranatuerin-1 (HP4)	KZ060483	3446	PIO12229	61	*Rana catesbeiana*	N/A	N/A	N/A	Rc-03r170621s168979	-	3446	1475-1107	1
ranatuerin-2PRc (HP2)	JQ511836	253	AFR43665	71	*Pseudacris regilla*	GFBS01116610	772	71	Rc-03r170621s5461	-	149396	114249-114211/108748-108668/100045-99394	3
ranatuerin-2RC	FJ830657	335	ACR84073	74	*Rana catesbeiana*	GFBS01229406	500	74	Rc-03r170621s59711; Rc-03r170621s128997	+/-	17230/ 5085	7179-7319, 11174-11256/ 4558-4326	3
ranatuerin-3RC	FJ830656	309	ACR84072	68	*Rana catesbeiana*	N/A	N/A	N/A	Rc-03r170621s223822; Rc-03r170621s584290	+/-	2611/ 1023	2144-2217/ 734-530	2
ranatuerin-3RC (HP8)	FJ830656	309	PIO09118	51	*Rana catesbeiana*	GFBS01228991	519	51	Rc-03r170621s584290	-	1023	1007-532	1
ranatuerin-4	BT081520	332	ACO51651	70	*Rana catesbeiana*	GFBS01229403	504	70	Rc-03r170621s71023; Rc-03r170621s251277	+/-	13560/ 2355	8821-8968, 10977-11059/ 2198-1964	3

[a]N/A indicates that the sequence found in the BART database was the same length as the *Rana catesbeiana* sequence already present in the NCBI database or that this sequence was not found in the BART database.
[b]This scaffold contained sequence that was 93% identical to the HP9 sequence but had 17 AA changes and an in-frame deletion of V36
[c]Translation begins with a V instead of M

A

```
AEI69698           MGLSATLWFLMGVAAGSMASPLLQWSEDDISVMALYSTDYYNKVSGEDVLYGLQENNTEY  60
Cathelicidin-AL    MGLSATFWFLMGLAASSMASPLLQWSEDDAAVMALYSADHYNKVSGEDVLYGLLENDTEY  60
                   ******:*****:**.************* :******:*:************* **:***

AEI69698           ITDEKSRFHQLSFPIQKTVCQKSDN-ALTDDCAFKEGGVVKSCTSYFFEEDDRDIIVVTC  119
Cathelicidin-AL    ITDEKSRFHQLSFPIQETVCQKSDNNAPTDDCAFKEGGVVKSCTSYFFEEDDRDIVVVNC  120
                   ****************:******** * ***************************:**.*

AEI69698           QSQDGHREHSRVRRSRRGRGGGRRGGSGGRGGRGGGGRSGAGSSIAGVGSRGGG--GGRHYA  179
Cathelicidin-AL    QSQDSHREHSRVRRSRSGRGGGGRGG-GGRGGSRGGSRSGSRSSIAGGGSRGGSRGGGTRYA  181
                   ****.*********** ***** *** *****  **.***: ***** *****.  ** :**
```

B

```
XP_018418722       MIPQLRKWMAIFVMCIVLIHQLEGAPMNSNDGSKTALRLRRMTPFWRGLSLRPVGASCRD  60
LEAP2              MTPQLRKWTAIFVICIVLIHQLEGAPMSNTAGSKTLLRLRRMTPFWRGLSLRPVGASCRD  60
                   * ****** ****:*************... **** ************************

XP_018418722       DTECLTRLCRNQRCSLKTFAD  81
LEAP2              DTECLTRLCRKERCSLKTFAD  81
                   **********::*********
```

Suppl. Fig. 1. Clustal omega alignments of putative AMP precursor sequences with their closest known AMP matches. A) Comparison of the *Amolops loloensis* Cathelicidin-AL sequence (NCBI Accession #AEI69698; top) with the corresponding *R. catesbeiana* Cathelicidin-AL sequence (bottom). B) Comparison of the *Nanorana parkeri* predicted LEAP2 sequence (NCBI Accession #XP_018418722; top) with the corresponding *R. catesbeiana* LEAP2 sequence (bottom). The conserved proteolytic cleavage site is bold and underlined. This cleavage site indicates the border for the N-terminal prepro sequence and the C-terminal mature sequence. The precursor peptide lengths are indicated to the right of each sequence. The dots represent conserved amino acid substitutions and asterisks indicate exact matches. Dashes were introduced to maximize sequence alignments. Further details regarding NCBI accession numbers are in **Suppl. Table 1**.
